# Weekly Teriparatide Treatment Decreases Microdamage in Tibial Trabecular Bone of Ovariectomized Cynomolgus Monkeys

**DOI:** 10.7759/cureus.92396

**Published:** 2025-09-15

**Authors:** Teppei Senda, Ken Iwata, Tasuku Mashiba, Mitsuru Saito, Masakazu Ishikawa

**Affiliations:** 1 Department of Orthopaedic Surgery, Faculty of Medicine, Kagawa University, Kita-gun, JPN; 2 Orthopaedic Surgery, Kagawa Saiseikai Hospital, Takamatsu, JPN; 3 Department of Orthopaedic Surgery, The Jikei University School of Medicine, Tokyo, JPN

**Keywords:** collagen cross-link, cynomolgus monkey, microdamage, osteoporosis, ovariectomy, teriparatide, tibia

## Abstract

Background/purpose: Osteoporosis is commonly treated with daily or weekly teriparatide, yet its impact on microdamage accumulation - a critical contributor to bone fragility - remains insufficiently evaluated in animal models. We evaluated the effects of weekly teriparatide on microdamage in tibial trabecular bone and examined its associations with bone mass, structure, turnover, and collagen cross-linking in ovariectomized (OVX) cynomolgus monkeys.

Methods: Seventy-seven adult female cynomolgus monkeys were randomized into four groups (n = 18-20 each): sham-operated, OVX + vehicle, and OVX + weekly teriparatide at 1.2 μg/kg or 6.0 μg/kg. After 18 months, proximal tibiae and iliac crest specimens were harvested. Tibial trabecular sections were assessed for histomorphometry, microdamage (crack density, crack surface density), and collagen cross-linking. Iliac crest samples were analyzed for bone turnover indices.

Results: The OVX group showed significantly higher microdamage accumulation compared to all other groups. Weekly teriparatide administration effectively prevented microdamage accumulation and improved collagen cross-link profile. Regression analysis revealed that reductions in microdamage correlated more strongly with decreased non-enzymatic pentosidine cross-links than with increases in trabecular bone volume or enzymatic cross-links.

Conclusions: Weekly teriparatide administration attenuates tibial trabecular bone microdamage by restoring collagen cross-link balance, independent of bone mass gain. This suggests that teriparatide enhances bone quality and resistance to fracture via modulation of bone matrix integrity.

## Introduction

Excessive secretion of endogenous parathyroid hormone (PTH), as observed in patients with hyperparathyroidism, enhances osteoclastic activity and promotes bone resorption, thereby contributing to the progression of osteoporosis [1]. In contrast, intermittent administration of PTH has been shown to exert anabolic effects on bone by stimulating the differentiation of osteoblastic progenitor cells, promoting the survival of mature osteoblasts, and transiently activating bone resorption. Notably, while osteoclastic activation is reversible upon cessation of PTH exposure, the increase in osteoblast number and activity persists, leading to sustained bone formation. Consequently, intermittent PTH therapy improves bone mass, architecture, and strength, and reduces the risk of fracture [2-5].

A once-weekly formulation of teriparatide acetate was developed in Japan [6] and has demonstrated clinical efficacy in patients with osteoporosis at high fracture risk. In addition to the weekly regimen, daily and twice-weekly formulations are also available, each of which has been reported to enhance bone mass based on human biopsy analyses and animal studies [7-10].

In a previous investigation, we demonstrated that weekly teriparatide administration in ovariectomized (OVX) cynomolgus monkeys enhanced bone quality in the lumbar spine by increasing trabecular bone volume and restoring collagen cross-linking balance [11]. However, its effects on bone microdamage - a critical determinant of bone fragility - remain unclear. While daily teriparatide administration has not been shown to reduce microdamage accumulation in iliac bone in clinical settings [12], this discrepancy raises the possibility that administration frequency may influence the balance between microdamage generation and repair.

Bone microdamage arises from mechanical loading and accumulates when the rate of damage exceeds the capacity for remodeling-mediated repair [13]. Given the anabolic and remodeling-stimulatory properties of teriparatide, it is plausible that the drug may contribute to microdamage mitigation through enhanced repair. Nevertheless, the impact of weekly teriparatide treatment on bone microdamage, particularly in large non-human primate models with bone turnover characteristics similar to humans, remains largely unexplored.

This study was designed with the primary objective of testing whether weekly administration of teriparatide reduces microdamage accumulation in proximal tibial trabecular bone compared with OVX + vehicle controls, with crack density (Cr.Dn) defined as the primary endpoint. The secondary objective was to examine the relationships between microdamage and bone volume (BV/TV), turnover, and collagen cross-links, particularly pentosidine, using only the OVX groups for analysis.

## Materials and methods

The animals, treatment protocols, and sample collection procedures used in this study were shared with those of our previously published study [9,11]. While the previous paper reported data from vertebral bone, the current study analyzes trabecular bone from the tibia. To avoid duplication, we have included only original data from the tibial site in this manuscript.

Animal model and study design

The animal model and study design used in this investigation have been described previously [9,11]. Briefly, 80 adult female cynomolgus monkeys (Macaca fascicularis), aged 12.0 ± 1.5 years (mean ± standard deviation), with closed epiphyseal lines, were procured from C.V. Universal Fauna (Jakarta Timur, Indonesia). Monkeys were housed under controlled environmental conditions (26 ± 2°C, 12-hour light/dark cycle) with ad libitum access to water and a standardized diet containing 1.4% calcium and 0.6% phosphorus (Harlan Sprague Dawley Inc., Indianapolis, IN, USA). Following a six-week acclimation period, 77 monkeys with stable health status and body weights ranging from 2.06 to 3.48 kg were randomly assigned to one of four groups (n = 18-20 per group): sham-operated, OVX + vehicle, and OVX + low-dose or high-dose teriparatide. Teriparatide acetate (human parathyroid hormone (1-34); Asahi Kasei Pharma Corp., Tokyo, Japan) was administered subcutaneously once weekly at a dose of either 1.2 or 6.0 μg/kg. Treatment was initiated immediately after sham surgery and one week after OVX. The OVX + vehicle group was administered subcutaneous injections of 0.1% saline with bovine serum albumin (BSA) on a weekly basis. All treatments continued for 18 months. For bone histomorphometry, all monkeys were double-labeled with intravenous calcein (4 mg/kg; Dojindo Laboratories, Kumamoto, Japan) on days 21 and seven before sacrifice. Euthanasia was performed using a lethal dose of pentobarbital sodium (Tokyo Chemical Industry Co., Ltd., Tokyo, Japan) in accordance with institutional animal care and use guidelines, as approved by the Animal Ethics Committee of our institution.

Sample preparation

The monkeys were euthanized at 18 months after experimentation, then left iliac crests and tibia were harvested, and adherent soft tissue was removed. We excluded 25 iliac crests and four tibia samples due to insufficient tissue volume. The final numbers of animals analyzed were as follows: tibia - SHAM n = 17, OVX n = 17, LOW n = 19, HIGH n = 20; iliac crest - SHAM n = 10, OVX n = 14, LOW n = 17, HIGH n = 12; and L3 vertebra - SHAM n = 19, OVX n = 18, LOW n = 19, HIGH n = 20. The iliac crest samples were preserved in 70% ethanol and embedded in a plastic resin based on methyl methacrylate. The acetabular fraction of the L3 vertebral body was cleaned to remove marrow, then frozen and powdered in liquid nitrogen, as described [9]. The tibia was fixed in 70% ethanol, stained with 1% basic fuchsin for 72 hours, and embedded in methacrylate-based plastic.

Bone histomorphometry analyses

We conducted dynamical and static histomorphometric analyses of iliac crest and tibial samples, respectively. Non-stained coronal tissue sections of the iliac crest, 5 μm thick, were prepared using a microtome. Tibial samples were cut into coronal sections with a diamond saw and polished to 100 µm thickness to evaluate bone structure and microdamage to trabecular bone.

**Figure 1:**
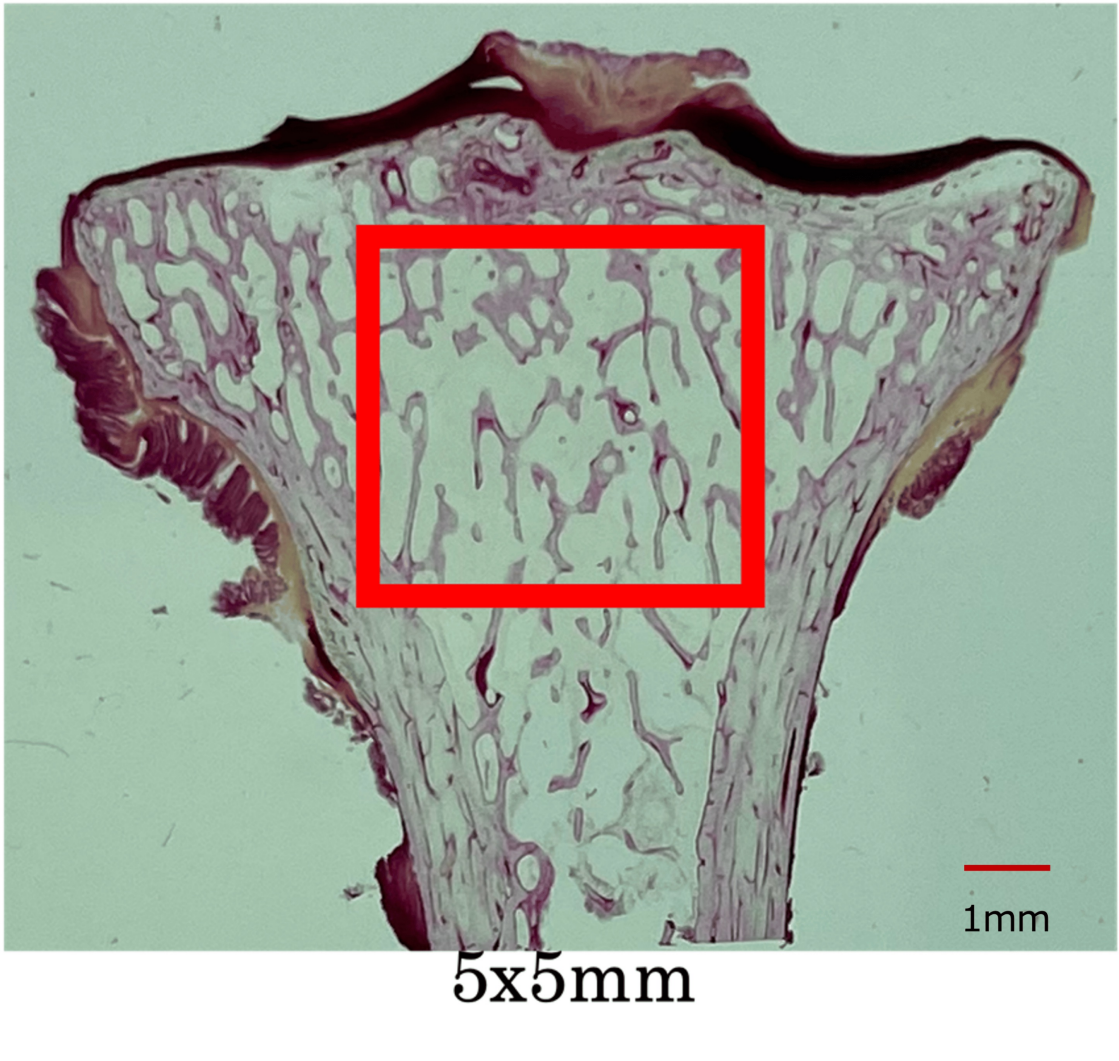
The square (5 × 5 mm) measurement area in the center of the tibial trabecular bone in the coronal section

We histomorphometrically analyzed bone samples using a semi-automated digitizing imaging system consisting of an optical or a grazing fluorescence microscope (magnification 100×) and a digitizing pad connected to a computer equipped with Bone Histomorphometric System software (System Supply, Nagano, Japan). Lamellar structure and trabecular packets were confirmed using polarization. A specialist in histomorphometry who was blinded to the group assignments measured all parameters. Trabecular bone structure and microdamage were assessed in 5 × 5 mm^2^ areas in the center of the bone (Figure 1).

**Figure 2:**
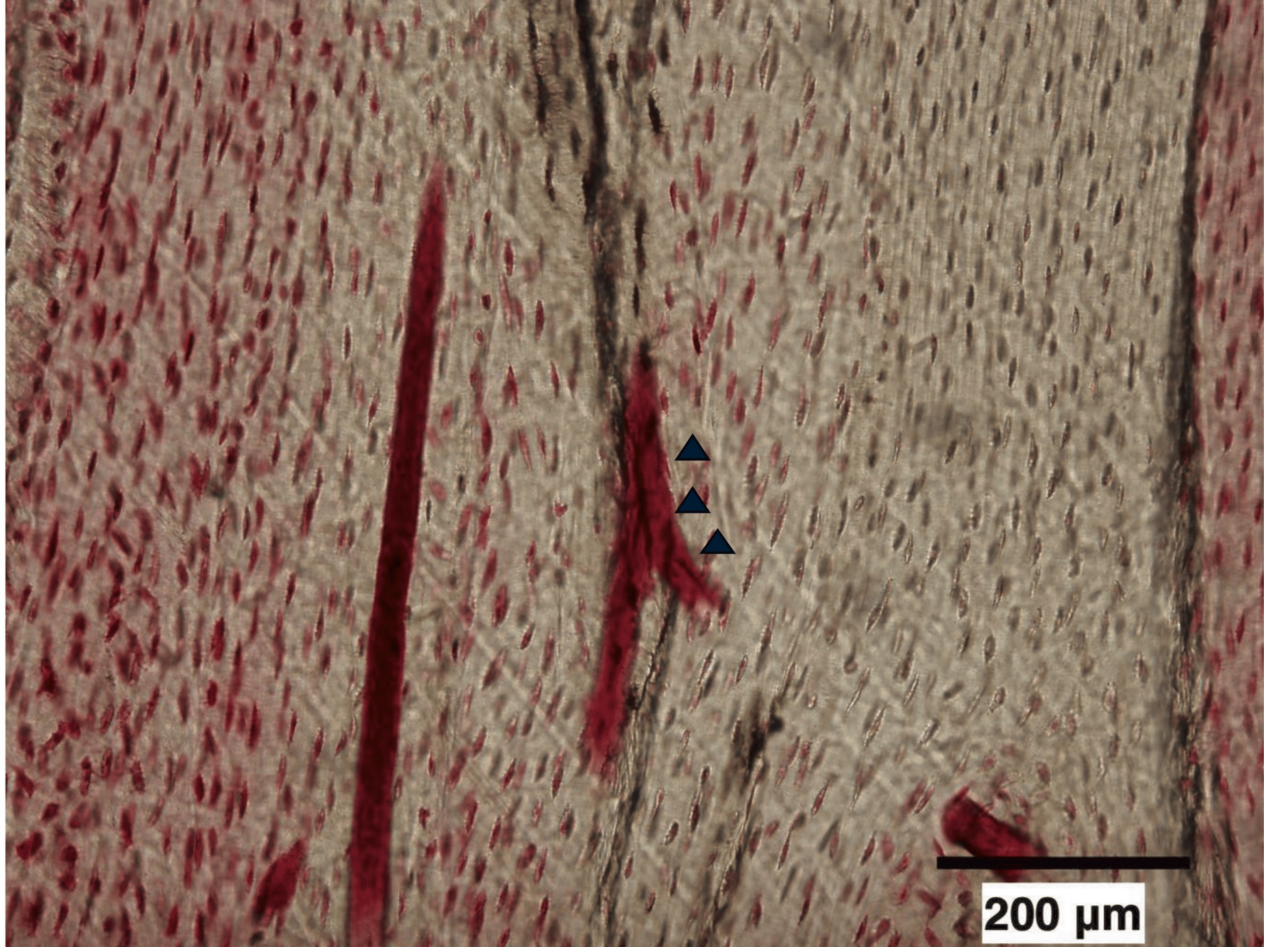
Microcrack in tibial trabecular bone stained with basic fuchsin (arrow). Original magnification, ×100.

Bone microdamage was identified as linear fuchsin-positive features within the bone matrix, visually distinguishable from physiological structures such as canaliculi and vascular channels. Although traditional definitions emphasize sharp linear borders, we also considered diffuse lamellar microcracks lacking well-defined edges to ensure sufficient inclusion for quantitative analysis (Figure 2) [14].

All bones with microdamage showed some depth and dye penetration into the crack wall [13,15]. We assessed the average crack length (Cr.Le, μm), crack density (Cr.Dn; Cr.N/B.Ar, #/mm^2^), and crack surface density (Cr.S.Dn; Cr.N × Cr.Le/B.Ar μm/mm^2^) as described. We analyzed form and structure by histomorphologically staining one tibial section per monkey.

We analyzed the structure of trabecular bone (Tb) by measuring the volume (BV/TV %), thickness (Tb.Th, μm), number per unit area (Tb.N/mm^2^), and separation (Tb.Sp, μm). Trabecular bone metrics were evaluated at the iliac crest as part of the histomorphometric analysis: single- (sLS/BS, %) and double- (dLS/BS, %) label surfaces, mineralization surface (MS/BS, %), mineral attachment rate (MAR, μm/day), bone formation rate (BFR/BS, μm^3^/μm^2^/year); and BFR per unit of bone volume (BFR/BV, %). The nomenclature and symbols used herein comply with those published by the Histomorphology Nomenclature Committee of the American Society for Bone Metabolism [16].

Measurement of collagen cross-links

We assessed collagen cross-linking as described [17]. Briefly, bone powder was demineralized in 50 mM Tris buffer (pH 7.4) containing 0.5 M EDTA at 4°C for 96 h, suspended in potassium phosphate buffer (pH 7.6) and reduced with sodium borohydride (Sigma-Aldrich, St. Louis, MO, USA) at 37°C. The samples were then passed through a Shimadzu LC9 high-performance liquid chromatography (HPLC) system equipped with a 0.9 × 10 cm, Aa pack-Na cation-exchange column (Jasco, Tokyo, Japan) and an online fluorescence flow monitor (RF10AXL; Shimadzu, Kyoto, Japan) to determine cross-linking and hydroxyproline content. The assumed weight of collagen was 7.5-fold that of measured hydroxyproline, based on a molecular weight of 300,000 Da [17]. Cross-linking was then calculated in terms of mol/mol collagen. The reducible immature cross-links dehydro-dihydroxylysino-norleucine (deH-DHLNL), dehydro-oxylysino-norleucine (deH-HLNL), and deydro-lysino-norleucine (deH-LNL) were identified and quantified in their reduced forms (DHLNL, HLNL, and LNL, respectively). The linear range of our HPLC system to determine amounts of enzymatic and non-enzymatic cross-links in bone samples is 0.2-600 pmol. Reducing cross-links and common amino acids such as hydroxyproline were detected by post-column derivatization with o-phthalaldehyde, and the non-reducing cross-links pyridinoline (Pyr), deoxypyridinoline (Dpyr) and pentosidine were detected by natural fluorescence. The enzymatic and non-enzymatic cross-linking units were measured in terms of mol/mol and mmol/mol of collagen, respectively. Pentosidine content was measured as described [18].

The collagen parameters measured at L3 were immature + mature pyridinium cross-links (DHNL + HLNL + LNL + Pyr + Dpyr, mol/collagen), mature cross-linking rate ([Pyr + Dpyr]/[DHNL + HLNL + LNL]), and pentosidine (mmol/collagen).

Statistical analysis

All data are expressed as means ± SD. Differences among four groups were assessed using one-way analysis of variance. Parameters explaining microcrack accumulation from trabecular bone structure, trabecular bone labeling, and collagen cross-linking in the three ovariectomized groups were examined using both Spearman rank correlation coefficients and Pearson correlation coefficients. Values with p < 0.05 were considered statistically significant. All data were statistically analyzed using EZR (Saitama Medical Center, Jichi Medical University, Saitama, Japan), which is a graphical user interface for R (The R Foundation for Statistical Computing, Vienna, Austria).

## Results

Trabecular bone structural parameters and microdamage parameters

The trabecular bone structural parameters (Table 1) did not significantly differ among the groups. The BV/TV tended to be slightly lower in the LOW than in the other groups, but the difference was not significant. None of the groups treated with teriparatide significantly differed.

**Table 1:** Structural parameters of tibial trabecular bone in four groups. Data are expressed as means ± standard deviation. No statistically significant differences were observed among groups. Statistical analysis was performed using one-way ANOVA. BV/TV, trabecular bone volume; Tb.N, trabecular number per unit area; Tb.Sp, trabecular one separation; Tb.Th, trabecular thickness.

	SHAM (n = 17)	OVX (n = 17)	LOW (n = 19)	HIGH (n = 20)
BV/TV (%)	21.9 ± 9.0	21.8 ± 7.5	17.9 ± 6.4	20.1 ± 6.2
Tb.Th (μm)	164.5 ± 47.0	163.5 ± 48.2	161.5 ± 40.9	164.6 ± 41.4
Tb.N (/mm)	1.31 ± 0.31	1.35 ± 0.32	1.11 ± 0.28	1.23 ± 0.28
Tb.Sp (μm)	651.0 ± 263.6	640.0 ± 246.5	800.0 ± 276.0	680.0 ± 169.2

The bone microdamage parameter Cr.Le did not significantly differ among the groups, whereas Cr.Dn and Sr.S.Dn were higher in the OVX, than in the other groups (p < 0.05; Table 2). Significant differences were not found among the other three parameters or between the low and high groups.

**Table 2:** Tibial bone microdamage parameters. Data are expressed as means ± standard abbreviation. *p < 0.05 vs. ovariectomy;determined by one-way ANOVA followed by Tukey’s post-hoc test. Cr.Dn, crack density; Cr.Le, average crack length; Cr.S.Dn, crack surface density.

	SHAM (n = 17)	OVX (n = 17)	LOW (n = 19)	HIGH (n = 20)
Cr.Le (μm)	48.8 ± 8.2	59.6 ± 10.9	67.0 ± 20.6	64.8 ± 18.8
Cr.Dn (/mm^2^)	1.22 ± 0.60*	3.48 ± 1.48	0.82 ± 0.58*	0.80 ± 0.45*
Cr.S.Dn (μm/mm^2^)	58.4 ± 26.3*	208.9 ± 96.6	56.4 ± 39.8*	51.7 ± 38.6*

Dynamic and collagen parameters

Dynamic histomorphometry parameters of the iliac crest (such as BFR/BS, MAR, and MS/BS) did not show significant differences among groups, as previously described [11]. Similarly, the enzymatic collagen cross-link content in L3 vertebrae was significantly increased in the teriparatide groups compared to OVX, while pentosidine levels were significantly decreased, in agreement with our earlier findings [11].

Correlations between microdamage and other parameters

**Figure 3:**
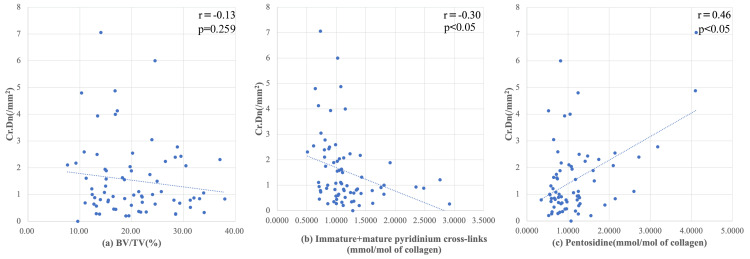
Regression analysis of microcrack density and other parameters. (a) Increased crack density (Cr.Dn) is not significantly associated with trabecular volume (BV/TV).(b) Numbers of enzymatic immature and mature collagen cross-links (IM + M) are decreased (c) whereas non-enzymatic cross-linked pentosidine has increased. r: Pearson’s correlation coefficient. p: p value; p < 0.05 considered significant.

We did not find any correlations between BV/TV and Cr.Dn (Figure 3A; r = -0.09, ρ = -0.09), whereas immature + mature pyridinium cross-linking (Figure 3B) slightly and negatively correlated with Cr.Dn (r = -0.3, ρ = -0.38). Among all parameters, pentosidine (Figure 3C) correlated most closely with increased Cr.Dn (r = 0.46, ρ = 0.25). Comparable results were obtained with Spearman’s rank correlation, supporting the consistency of the findings.

## Discussion

This study evaluated the effects of weekly teriparatide administration on trabecular bone structure, metabolism, and microdamage in OVX cynomolgus monkeys, a well-established non-human primate model of postmenopausal osteoporosis. Despite the absence of significant improvements in trabecular bone structural parameters, weekly teriparatide treatment significantly reduced the accumulation of bone microdamage. These findings suggest that the therapeutic benefits of teriparatide may extend beyond bone mass enhancement, implicating alternative mechanisms in the preservation of bone integrity. Although previous studies on bone microdamage are limited [11,12], the present study provides a comprehensive evaluation of the effects of teriparatide on microdamage accumulation and repair.

Teriparatide promotes the repair of bone microdamage through stimulation of bone remodeling; thus, its ability to reduce microdamage accumulation may be attributed to increased bone turnover [13,15,19]. In the present study, microdamage accumulation was significantly greater in the OVX group than in both the sham and OVX + teriparatide groups. Estrogen deficiency following ovariectomy leads to increased bone resorption, resulting in osteoporosis with a hypermetabolic turnover state, which primarily manifests as loss of trabecular bone mass [20-22].

Although we presumed that the OVX group represented a model of postmenopausal osteoporosis with elevated bone turnover, no significant differences in trabecular bone structural parameters were observed among the four groups. This finding suggests that, despite presumed remodeling, the rate of microdamage generation exceeded the rate of repair in the OVX group. The observed reductions in Cr.Dn and Cr.S.Dn in the sham and OVX + teriparatide groups likely reflect both decreased microdamage generation and enhanced repair capacity. Estrogen deficiency may contribute to increased microdamage accumulation and delayed repair due to compromised bone quality, whereas PTH treatment appears to enhance both bone strength and the capacity for remodeling-based repair.

Furthermore, the hypermetabolic turnover induced by ovariectomy in cynomolgus monkeys may be transient [23], with turnover rates returning to baseline within 18 months postoperatively. The absence of significant differences in bone remodeling indices at the time of analysis suggests that the observed reductions in microdamage in the teriparatide group were not due to persistent turnover elevation, but rather to improved remodeling efficiency and bone matrix quality.

Weekly PTH administration has been shown to preserve cortical bone volume and tissue mineral density, while increasing cortical thickness and overall bone strength [24]. Although its effects on trabecular bone have been characterized as moderate, they occur without reductions in cortical mineral density. In a previous report, no significant changes in Tb.Th of the distal tibia were observed after eight months of weekly PTH treatment. Our current findings are consistent with these observations, as we similarly observed no significant differences in Tb.Th following weekly teriparatide administration.

A previous study in mice reported that less frequent administration of teriparatide induces bone formation through both remodeling and mini-modeling processes [25]. This mechanism may explain the unique action of teriparatide, wherein bone formation via modeling occurs without a concomitant increase in bone resorption. Weekly administration of teriparatide may therefore open an “anabolic window,” characterized by a temporary enhancement of bone formation that is not accompanied by increased resorption [26]. Based on this mechanism, we speculate that weekly teriparatide promotes new bone formation and facilitates the repair of microdamage by transiently stimulating bone remodeling activity.

Collagen fibers in the bone matrix contain both physiological and age-related cross-links. The latter are primarily formed by advanced glycation end-products (AGEs), which accumulate over time through non-enzymatic glycation reactions, with pentosidine being a representative compound. Although these cross-links are typically associated with highly calcified bone matrix, they are often found in greater abundance in poorly mineralized regions [9]. Elevated levels of pentosidine have been reported to impair bone material properties and reduce bone strength [27]. Pentosidine accumulation is elevated after ovariectomy, presumably driven by oxidative mechanisms linked to estrogen withdrawal, and this occurs even when bone turnover remains high [28]. In contrast, increases in immature and mature pyridinium cross-links - products of enzymatic collagen cross-linking - are considered physiological and contribute positively to bone strength. This study revealed that a rise in enzymatic collagen cross-links (immature and mature pyridinium) combined with a reduction in pentosidine levels [29,30] was linked to a decrease in Cr.Dn, suggesting enhanced bone material properties. In contrast, the OVX group, which exhibited elevated pentosidine and reduced enzymatic cross-linking, showed increased microdamage, reflecting compromised bone matrix strength.

We previously reported a significant negative correlation between pentosidine content and lumbar spine stiffness [9]. In the present study, pentosidine content demonstrated the strongest association with increased microdamage accumulation. These findings suggest that elevated pentosidine levels contribute to bone fragility by promoting the accumulation of bone microdamage.

The low and high doses of teriparatide administered in this study were based on clinical dosing standards in Japan - corresponding to the standard weekly dose for osteoporosis as per the 1994 FDA guidelines, and a five-fold higher dose, respectively. Notably, there were no significant differences in microdamage accumulation between the low-dose and high-dose groups, indicating that the clinical dose of weekly teriparatide was sufficient to prevent microdamage. Based on these findings, we propose that weekly teriparatide administration at the approved clinical dose is an effective therapeutic strategy for reducing bone fragility in osteoporotic patients.

This study has several limitations. First, we did not assess the mechanical strength of the proximal tibia; therefore, we were unable to directly correlate microdamage with mechanical properties. Although previous studies have demonstrated significant associations between weekly teriparatide treatment, collagen cross-link profiles, and mechanical strength, our findings suggest that the observed reduction in pentosidine may be clinically relevant in terms of reducing microdamage accumulation, as indicated by decreased Cr.Dn and Cr.S.Dn.

Second, dynamic bone turnover parameters were evaluated only in the iliac crest and not in the tibia. This was due to the original study design initiated several years ago, in which fluorochrome labeling was performed immediately prior to euthanasia to preserve labeling integrity. If dynamic parameters had been assessed in the tibia at the time of sacrifice, more accurate insights into the relationship between turnover and microdamage accumulation in the same anatomical region may have been obtained.

Third, microdamage was assessed in specimens stored in 70% ethanol for several years. Long-term storage may have altered bone material properties and potentially increased artifact microdamage due to specimen sectioning. Finally, since microdamage was analyzed only postmortem, we could not determine whether teriparatide reduced microdamage by enhancing repair or suppressing its formation. Longitudinal assessment during treatment would be necessary to elucidate the time course and mechanism of microdamage reduction.

We have also added a note in the Limitations section explicitly stating that the comparison between cynomolgus monkey and human dosing is based only on approximate weight-based conversion and should be interpreted with caution.

Despite these limitations, in OVX cynomolgus monkeys, weekly teriparatide treatment resulted in a notable reduction of microdamage within the trabecular bone of the proximal tibia. This reduction was associated with decreased pentosidine levels, independent of BV/TV or architectural improvement. These results suggest that weekly teriparatide improves bone matrix quality not only in the lumbar spine but also in the tibia, thereby exerting potent anti-fracture effects. 

## Conclusions

While further investigation is required to determine the translational relevance of these findings to human osteoporosis treatment, our data support the therapeutic potential of weekly teriparatide in enhancing bone quality and reducing fragility.
